# Evaluation of ChatGPT-Generated Differential Diagnosis for Common Diseases With Atypical Presentation: Descriptive Research

**DOI:** 10.2196/58758

**Published:** 2024-06-21

**Authors:** Kiyoshi Shikino, Taro Shimizu, Yuki Otsuka, Masaki Tago, Hiromizu Takahashi, Takashi Watari, Yosuke Sasaki, Gemmei Iizuka, Hiroki Tamura, Koichi Nakashima, Kotaro Kunitomo, Morika Suzuki, Sayaka Aoyama, Shintaro Kosaka, Teiko Kawahigashi, Tomohiro Matsumoto, Fumina Orihara, Toru Morikawa, Toshinori Nishizawa, Yoji Hoshina, Yu Yamamoto, Yuichiro Matsuo, Yuto Unoki, Hirofumi Kimura, Midori Tokushima, Satoshi Watanuki, Takuma Saito, Fumio Otsuka, Yasuharu Tokuda

**Affiliations:** 1 Department of General Medicine, Chiba University Hospital, Chiba, Japan; 2 Department of Community-Oriented Medical Education, Chiba University Graduate School of Medicine, Chiba, Japan; 3 Department of Diagnostic and Generalist Medicine, Dokkyo Medical University, Tochigi, Japan; 4 Department of General Medicine, Dentistry and Pharmaceutical Sciences, Okayama University Graduate School of Medicine, Okayama, Japan; 5 Department of General Medicine, Saga University Hospital, Saga, Japan; 6 Department of General Medicine, Juntendo University Hospital Faculty of Medicine, Tokyo, Japan; 7 Integrated Clinical Education Center Hospital Integrated Clinical Education, Kyoto University Hospital, Kyoto, Japan; 8 Department of General Medicine and Emergency Care, Toho University School of Medicine, Tokyo, Japan; 9 Center for Preventive Medical Sciences, Chiba University, Chiba, Japan; 10 Tama Family Clinic, Kanagawa, Japan; 11 Department of General Medicine, Awa Regional Medical Center, Chiba, Japan; 12 Department of General Medicine, National Hospital Organization Kumamoto Medical Center, Kumamoto, Japan; 13 Department of Neurology, University of Utah, Salt Lake City, UT, United States; 14 Department of Internal Medicine, Mito Kyodo General Hospital, Ibaraki, Japan; 15 Tokyo Metropolitan Hiroo Hospital, Tokyo, Japan; 16 Department of Molecular and Human Genetics, Baylor College of Medicine, Houston, TX, United States; 17 Division of General Medicine, Nerima Hikarigaoka Hospital, Tokyo, Japan; 18 Department of General Medicine, Nara City Hospital, Nara, Japan; 19 Department of General Internal Medicine, St. Luke’s International Hospital, Tokyo, Japan; 20 Division of General Medicine, Center for Community Medicine, Jichi Medical University, Tochigi, Japan; 21 Department of Clinical Epidemiology and Health Economics, The Graduate School of Medicine, The University of Tokyo, Tokyo, Japan; 22 Department of General Internal Medicine, Iizuka Hospital, Fukuoka, Japan; 23 Saga Medical Career Support Center, Saga University Hospital, Saga, Japan; 24 Department of Emergency and General Medicine, Tokyo Metropolitan Tama Medical Center, Tokyo, Japan; 25 Muribushi Okinawa Center for Teaching Hospitals, Okinawa, Japan; 26 Tokyo Foundation for Policy Research, Tokyo, Japan

**Keywords:** atypical presentation, ChatGPT, common disease, diagnostic accuracy, diagnosis, patient safety

## Abstract

**Background:**

The persistence of diagnostic errors, despite advances in medical knowledge and diagnostics, highlights the importance of understanding atypical disease presentations and their contribution to mortality and morbidity. Artificial intelligence (AI), particularly generative pre-trained transformers like GPT-4, holds promise for improving diagnostic accuracy, but requires further exploration in handling atypical presentations.

**Objective:**

This study aimed to assess the diagnostic accuracy of ChatGPT in generating differential diagnoses for atypical presentations of common diseases, with a focus on the model’s reliance on patient history during the diagnostic process.

**Methods:**

We used 25 clinical vignettes from the *Journal of Generalist Medicine* characterizing atypical manifestations of common diseases. Two general medicine physicians categorized the cases based on atypicality. ChatGPT was then used to generate differential diagnoses based on the clinical information provided. The concordance between AI-generated and final diagnoses was measured, with a focus on the top-ranked disease (top 1) and the top 5 differential diagnoses (top 5).

**Results:**

ChatGPT’s diagnostic accuracy decreased with an increase in atypical presentation. For category 1 (C1) cases, the concordance rates were 17% (n=1) for the top 1 and 67% (n=4) for the top 5. Categories 3 (C3) and 4 (C4) showed a 0% concordance for top 1 and markedly lower rates for the top 5, indicating difficulties in handling highly atypical cases. The *χ*^2^ test revealed no significant difference in the top 1 differential diagnosis accuracy between less atypical (C1+C2) and more atypical (C3+C4) groups (*χ*²_1_=2.07; n=25; *P*=.13). However, a significant difference was found in the top 5 analyses, with less atypical cases showing higher accuracy (*χ*²_1_=4.01; n=25; *P*=.048).

**Conclusions:**

ChatGPT-4 demonstrates potential as an auxiliary tool for diagnosing typical and mildly atypical presentations of common diseases. However, its performance declines with greater atypicality. The study findings underscore the need for AI systems to encompass a broader range of linguistic capabilities, cultural understanding, and diverse clinical scenarios to improve diagnostic utility in real-world settings.

## Introduction

For the past decade, medical knowledge and diagnostic techniques have expanded worldwide, becoming more accessible with remarkable advancements in clinical testing and useful reference systems [1]. Despite these advancements, misdiagnosis significantly contributes to mortality, making it a noteworthy public health issue [2,3]. Studies have revealed discrepancies between clinical and postmortem autopsy diagnoses in at least 25% of cases, with diagnostic errors contributing to approximately 10% of deaths and to 6%‐17% of hospital adverse events [4-8]. The significance of atypical presentations as a contributor to diagnostic errors is especially notable, with recent findings suggesting that such presentations are prevalent in a substantial portion of outpatient consultations and are associated with a higher risk of diagnostic inaccuracies [9]. This underscores the persistent challenge in diagnosing patients correctly due to the variability in disease presentation and due to the reliance on medical history, which is the basis for approximately 80% of the medical diagnosis [10,11].

The advent of artificial intelligence (AI) in health care, particularly through natural language processing (NLP) models such as generative pre-trained transformers (GPTs), has opened new avenues in medical diagnosis [12]. Recent studies on AI medical diagnosis across various specialties—including neurology [13], dermatology [14], radiology [15], and pediatrics [16]—have shown promising results and improved diagnostic accuracy, efficiency, and safety. Among these developments, GPT-4, a state-of-the-art AI model developed by OpenAI, has demonstrated remarkable capabilities in understanding and processing medical language, significantly outperforming its predecessors in medical knowledge assessments and potentially transforming medical education and clinical decision support systems [12,17].

**Figure 1.**
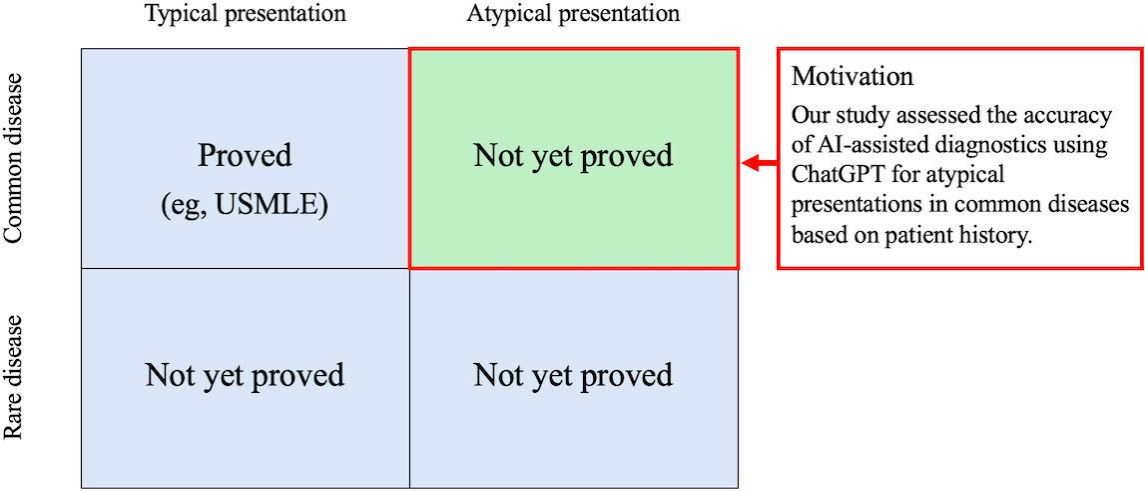
Study motivation. AI: artificial intelligence; USMLE: United States Medical Licensing Examination.

Notably, one study found that ChatGPT (OpenAI) could pass the United States Medical Licensing Examination (USMLE), highlighting its potential in medical education and medical diagnosis [18,19]. Moreover, in controlled settings, ChatGPT has shown over 90% accuracy in diagnosing common diseases with typical presentations based on chief concerns and patient history [20]. However, while research has examined the diagnostic accuracy of AI chatbots, including ChatGPT, in generating differential diagnoses for complex clinical vignettes derived from general internal medicine (GIM) department case reports, their diagnostic accuracy in handling atypical presentations of common diseases remains less explored [21,22]. There has been a notable study aimed at evaluating the accuracy of the differential diagnosis lists generated by both third- and fourth-generation ChatGPT models using case vignettes from case reports published by the Department of General Internal Medicine of Dokkyo Medical University Hospital, Japan. ChatGPT with GPT-4 was found to achieve a correct diagnosis rate in the top 10 differential diagnosis lists, top 5 lists, and top diagnoses of 83%, 81%, and 60%, respectively—rates comparable to those of physicians. Although the study highlights the potential of ChatGPT as a supplementary tool for physicians, particularly in the context of GIM, it also underlines the importance of further investigation into the diagnostic accuracy of ChatGPT with atypical disease presentations (Figure 1). Given the crucial role of patient history in diagnosis and the inherent variability in disease presentation, our study expands upon this foundation to assess the accuracy of ChatGPT in diagnosing common diseases with atypical presentations [23].

More specifically, this study aims to evaluate the hypothesis that the diagnostic accuracy of AI, exemplified by ChatGPT, declines when dealing with atypical presentations of common diseases. We hypothesize that despite the known capabilities of AI in recognizing typical disease patterns, its performance will be significantly challenged when presented with clinical cases that deviate from these patterns, leading to reduced diagnostic precision. Consequently, this study seeks to systematically assess this hypothesis and explore its implications for the integration of AI in clinical practice. By exploring the contribution of AI-assisted medical diagnoses to common diseases with atypical presentations and patient history, the study assesses the accuracy of ChatGPT in reaching a clinical diagnosis based on the medical information provided. By reevaluating the significance of medical information, our study contributes to the ongoing discourse on optimizing diagnostic processes—both conventional and AI assisted.

## Methods

### Study Design, Settings, and Participants

This study used a series of 25 clinical vignettes from a special issue of the *Journal of Generalist Medicine*, a Japanese journal, published on March 5, 2024. These vignettes, which exemplify atypical presentations of common diseases, were selected for their alignment with our research aim to explore the impact of atypical disease presentations in AI-assisted diagnosis. The clinical vignettes were derived from real patient cases and curated by an editorial team specializing in GIM, with final edits by KS. Each case included comprehensive details such as age, gender, chief concern, medical history, medication history, current illness, and physical examination findings, along with the ultimate and initial misdiagnoses.

**Figure 2.**
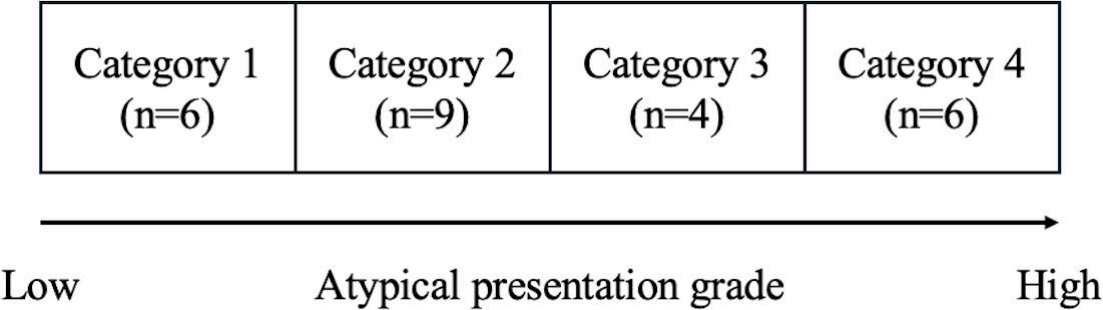
Categories of common diseases with atypical presentations (n=25).

An expert panel comprising 2 general medicine and medical education physicians, T Shimizu and Y Otsuka, initially reviewed these cases. After deliberation, they selected all 25 cases that exemplified atypical presentations of common diseases. Subsequently, T Shimizu and Y Otsuka evaluated their degree of atypicality and categorized them into 4 distinct levels, using the following definition as a guide: “Atypical presentations have a shortage of prototypical features. These can be defined as features that are most frequently encountered in patients with the disease, features encountered in advanced presentations of the disease, or simply features of the disease commonly listed in medical textbooks. Atypical presentations may also have features with unexpected values” [24]. Category 1 was assigned to cases that were closest to the typical presentations of common diseases, whereas category 4 was designated for those that were markedly atypical. In instances where T Shimizu and Y Otsuka did not reach consensus, a third expert, KS, was consulted. Through collaborative discussions, the panel reached a consensus on the final category for each case, ensuring a systematic and comprehensive evaluation of the atypical presentations of common diseases (Figure 2).

Our analysis was conducted on March 12, 2024, using ChatGPT’s proficiency in Japanese. The language processing was enabled by the standard capabilities of the ChatGPT model, requiring no additional adaptation or programming by our team. We exclusively used text-based input for the generative AI, excluding tables or images to maintain a focus on linguistic data. This approach is consistent with the typical constraints of language-based AI diagnostic tools. Inputs to ChatGPT consisted of direct transcriptions of the original case reports in Japanese, ensuring the authenticity of the medical information was preserved. We measured the concordance between AI-generated differential diagnoses and the vignettes’ final diagnoses, as well as the initial misdiagnoses. Our investigation entailed inputting clinical information—including medical history, physical examination, and laboratory data—into ChatGPT, followed by posing this request: “List of differential diagnoses in order of likelihood, based on the provided vignettes’ information,” labeled as “GAI [generative AI] differential diagnoses.”

### Data Collection and Measurements

**Figure 3.**
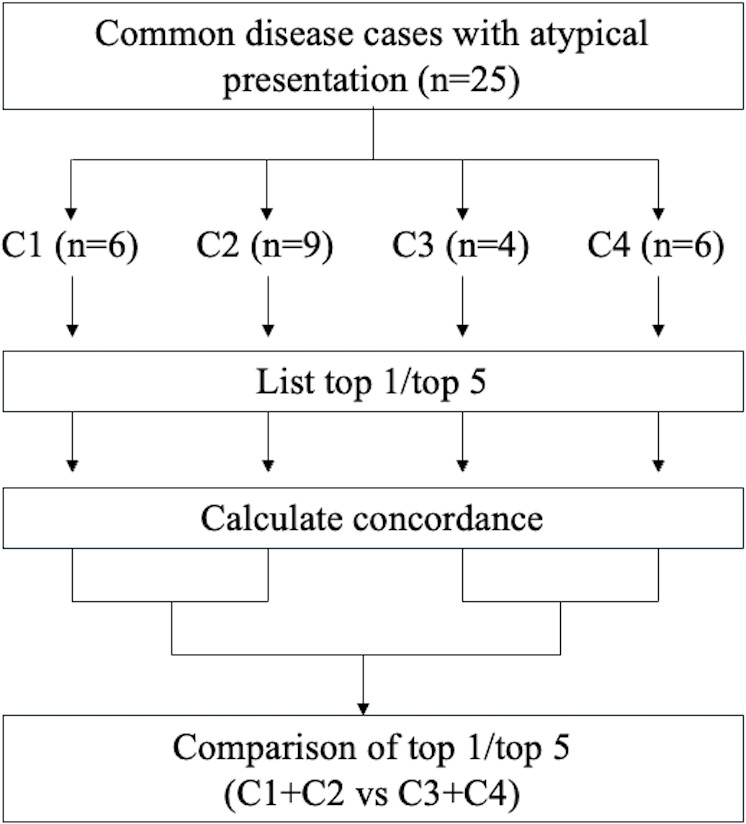
Study flow. C: category.

We assigned the correct diagnosis for each of these 25 cases as “final diagnosis.” We then used ChatGPT to generate differential diagnoses (“GAI differential diagnoses”). For each case, ChatGPT was prompted to create a list of differential diagnoses. Patient information was provided in full each time, without incremental inputs. The concordance rate between “final diagnosis,” “misdiagnosis,” and “GAI differential diagnoses” was then assessed. To extract a list of diagnoses from ChatGPT, we concluded each input session with the phrase “List of differential diagnoses in order of likelihood, based on the provided vignettes’ information.” We measured the percentage at which the final diagnosis or misdiagnosis was included in the top-ranked disease (top 1) and within the top 5 differential diagnoses (top 5) generated by ChatGPT (Figure 3).

### Data Analysis

Two board-certified physicians working in the medical diagnostic department of our facility judged the concordance between the AI-proposed diagnoses and the final diagnosis. The 2 physicians are GIM board–certified. The number of years after graduation of the physicians was 7 and 17, respectively. A diagnosis was considered to match if the 2 physicians agreed to the concordance. We measured the interrater reliability with the κ coefficient (0.8‐1.0=almost perfect; 0.6‐0.8=substantial; 0.4‐0.6=moderate; and 0.2‐0.4=fair) [25]. To further analyze the accuracy of the top 1 and top 5 diagnoses, we used the *χ*² or Fisher exact test, as appropriate. Statistical analyses were conducted using SPSS Statistics (version 26.0; IBM Corp) with the level of significance set at *P*<.05.

### Ethics Approval

Our research did not involve humans, medical records, patient information, observations of public behaviors, or secondary data analyses; thus, it was exempt from ethical approval, informed consent requirements, and institutional review board approval. Additionally, as no identifying information was included, the data did not need to be anonymized or deidentified. We did not offer any compensation because there were no human participants in the study.

## Results

The 25 clinical vignettes comprised 11 male and 14 female patients, with ages ranging from 21 to 92 years. All individuals were older than 20 years, and 8 were older than 65 years. Table 1, Multimedia Appendix 1, and Multimedia Appendix 2 present these results. The correct final diagnosis listed in the *Journal of Generalist Medicine* clinical vignette as a common disease presenting atypical symptoms (labeled as “final diagnosis”) showed that “GAI differential diagnoses” and “final diagnosis” coincided in 12% (3/12) of cases within the first list of differential diagnoses, while “GAI differential diagnoses” and “final diagnosis” had a concordance rate of 44% (11/25) in 5 differential diagnoses. The interrater reliability was substantial (Cohen κ=0.84).

The analysis of the concordance rates between the “GAI differential diagnoses” generated by ChatGPT and the “final diagnosis” from the *Journal of Generalist Medicine* revealed distinct patterns across the 4 categories of atypical presentations (Table 2). For the top 1 differential diagnosis, that is, category 1 (C1) cases, which were closest to a typical presentation, the concordance rate was 7% (n=1), whereas category 2 (C2) cases exhibited a slightly higher rate of 22% (n=2). Remarkably, categories 3 (C3) and 4 (C4), which represent more atypical cases, demonstrated no concordance (0%) in the top 1 differential diagnosis.

When the analysis was expanded to the top 5 differential diagnoses, the concordance rates varied across categories. C1 cases showed a significant increase in concordance, to 67% (n=4), indicating better performance of the “GAI differential diagnoses” when considering a broader range of possibilities. C2 cases had a concordance rate of 44% (n=4), followed by C3 cases at 25% (n=1) and C4 cases at 17% (n=1).

To assess the diagnostic accuracy of ChatGPT across varying levels of atypical presentations, we used the *χ*^2^ test. Specifically, we compared the frequency of correct diagnoses in the top 1 and top 5 differential diagnoses provided by ChatGPT for cases categorized as C1+C2 (less atypical) versus C3+C4 (more atypical). For the top 1 differential diagnosis, there was no statistically significant difference in the number of correct diagnoses between the less atypical (C1+C2) and more atypical (C3+C4) groups (*χ*²_1_=2.07; n=25; *P*=.13). However, when expanding the analysis to the top 5 differential diagnoses, we found a statistically significant difference, with the less atypical group (C1+C2) demonstrating a higher number of correct diagnoses compared to the more atypical group (C3+C4*) (χ*²_1_=4.01; n=25; *P*=.048).

**Table 1.** List of answers and diagnoses provided by ChatGPT. Category 1 was closest to typical, and category 4 was most atypical.

Case	Age (years)	Gender	Final diagnosis^a^	Category	GAI^b^ diagnosis rank^c^
1	34	F	Caffeine intoxication	1	0
2	40	F	Asthma	1	1
3	55	F	Obsessive-compulsive disorder	1	3
4	58	M	Drug-induced enteritis	1	3
5	38	F	Cytomegalovirus infection	1	3
6	29	M	Acute HIV infection	1	5
7	62	M	Cardiogenic cerebral embolism	2	1
8	70	M	Cervical epidural hematoma	2	0
9	70	F	Herpes zoster	2	0
10	86	F	Hemorrhagic gastric ulcer	2	0
11	77	M	Septic arthritis	2	3
12	78	F	Compression fracture	2	0
13	45	M	Infective endocarditis	2	0
14	21	F	Ectopic pregnancy	2	1
15	55	F	Non-ST elevation myocardial infarction	2	2
16	54	F	Hypoglycemia	3	0
17	77	F	Giant cell arteritis	3	0
18	60	M	Adrenal insufficiency	3	4
19	38	F	Generalized anxiety disorder	3	0
20	24	F	Graves disease	4	4
21	31	M	Acute myeloblastic leukemia	4	0
22	76	F	Elderly onset rheumatoid arthritis	4	0
23	45	M	Appendicitis	4	0
24	92	M	Rectal cancer	4	0
25	60	M	Acute aortic dissection	4	0

a Final diagnosis indicates the final correct diagnosis listed in the *Journal of Generalist Medicine* clinical vignette as common disease presenting atypical symptoms.

b GAI: generative artificial intelligence.

c GAI diagnosis rank indicates the high-priority differential diagnosis rank generated by ChatGPT.

**Table 2.** Concordance rates of artificial intelligence–generated differential diagnoses by atypicality category. Category (C) 1 was closest to typical, and C4 was most atypical.

Category	Rank 1 diagnoses, n	Rank 2 diagnoses, n	Rank 3 diagnoses, n	Rank 4 diagnoses, n	Rank 5 diagnoses, n	Misdiagnoses, n	Top 1, %	Top 5, %
C1	1	0	3	0	0	2	17	67
C2	2	1	1	0	0	5	22	44
C3	0	0	0	1	0	3	0	25
C4	0	0	0	1	0	5	0	17

## Discussion

### Principal Findings

This study provides insightful data on the performance of ChatGPT in diagnosing common diseases with atypical presentations. Our findings offer a nuanced view of the capacity of AI-driven differential diagnoses across varying levels of atypicality. In the analysis of the concordance rates between “GAI differential diagnoses” and “final diagnosis,” we observed a decrease in diagnostic accuracy as the degree of atypical presentation increased.

The performance of ChatGPT in C1 cases, which are the closest to typical presentations, was moderately successful, with a concordance rate of 17% for the top 1 diagnosis and 67% within the top 5. This suggests that when the disease presentation closely aligns with the typical characteristics known to the model, ChatGPT is relatively reliable at identifying a differential diagnosis list that coincides with the final diagnosis. However, the utility of ChatGPT appears to decrease as atypicality increases, as evidenced by the lower concordance rates in C2, and notably more so in C3 and C4, where the concordance rates for the top 1 diagnosis fell to 0%. Similar challenges were observed in another 2024 study [26], where the diagnostic accuracy of ChatGPT varied depending on the disease etiology, particularly in differentiating between central nervous system and non–central nervous system tumors.

It is particularly revealing that in the more atypical presentations of common diseases (C3 and C4), the AI struggled to provide a correct diagnosis, even within the top 5 differential diagnoses, with concordance rates of 25% and 17%, respectively. These categories highlight the current limitations of AI in medical diagnosis when faced with cases that deviate significantly from the established patterns within its training data [27].

By leveraging the comprehensive understanding and diagnostic capabilities of ChatGPT, this study aims to reevaluate the significance of patient history in AI-assisted medical diagnosis and contribute to optimizing diagnostic processes [28]. Our exploration of ChatGPT’s performance in processing atypical disease presentations not only advances our understanding of AI’s potential in medical diagnosis [23] but also underscores the importance of integrating advanced AI technologies with traditional diagnostic methodologies to enhance patient care and reduce diagnostic errors.

The contrast in performance between the C1 and C4 cases can be seen as indicative of the challenges AI systems currently face with complex clinical reasoning requiring pattern recognition. Atypical presentations can include uncommon symptoms, rare complications, or unexpected demographic characteristics, which may not be well represented in the data sets used to train the AI systems [29]. Furthermore, these findings can inform the development of future versions of AI medical diagnosis systems and guide training curricula to include a broader spectrum of atypical presentations.

This study underscores the importance of the continued refinement of AI medical diagnosis systems, as highlighted by the recent advances in AI technologies and their applications in medicine. Studies published in 2024 [30-32] provide evidence of the rapidly increasing capabilities of large language models (LLMs) like GPT-4 in various medical domains, including oncology, where AI is expected to significantly impact precision medicine [30]. The convergence of text and image processing, as seen in multimodal AI models, suggests a qualitative leap in AI’s ability to process complex medical information, which is particularly relevant for our findings on AI-assisted medical diagnostics [30]. These developments reinforce the potential of AI tools like ChatGPT in bridging the knowledge gap between machine learning developers and practitioners, as well as their role in simplifying complex data analyses in medical research and practice [31]. However, as these systems evolve, it is crucial to remain aware of their limitations and the need for rigorous verification processes to mitigate the risk of errors, which can have significant implications in clinical settings [32]. This aligns with our observation of decreased diagnostic accuracy in atypical presentations and the necessity for cautious integration of AI into clinical practice. It also points to the potential benefits of combining AI with human expertise to compensate for current AI limitations and enhance diagnostic accuracy [33].

Our research suggests that while AI, particularly ChatGPT, shows promise as a supplementary tool for medical diagnosis, reliance on this technology should be balanced with expert clinical judgment, especially in complex and atypical cases [28,29]. The observed concordance rate of 67% for C1 cases indicates that even when not dealing with extremely atypical presentations, cases with potential pitfalls may result in AI medical diagnosis accuracy lower than the 80%‐90% estimated by existing studies [10,11]. This revelation highlights the need for cautious integration of AI in clinical settings, acknowledging that its diagnostic capabilities, while robust, may still fall short in certain scenarios [34,35].

### Limitations

Despite the strengths of our research, the study has certain limitations that must be noted when contextualizing our findings. First, the external validity of the results may be limited, as our data set comprises only 25 clinical vignettes sourced from a special issue of the *Journal of Generalist Medicine*. While these vignettes were chosen for their relevance to the study’s hypothesis on atypical presentations of common diseases, the size of the data set and its origin as mock scenarios rather than real patient data may limit the generalizability of our findings. This sample size may not adequately capture the variability and complexities typically encountered in broader clinical practice and thus might not be sufficient to firmly establish statistical generalizations. This limitation is compounded by the exclusion of pediatric vignettes, which narrows the demographic range of our findings and potentially reduces their applicability across diverse age groups.

Second, ChatGPT’s current linguistic capabilities predominantly cater to English, presenting significant barriers to patient-provider interactions that may occur in other languages. This raises concerns about the potential for miscommunication and subsequent misdiagnosis in non-English medical consultations. This underscores the essential need for future AI models to exhibit a multilingual capacity that can grasp the subtleties inherent in various languages and dialects, as well as the cultural contexts within which they are used.

Finally, the diagnostic prioritization process of ChatGPT did not always align with clinical probabilities, potentially skewing the perceived effectiveness of the AI model. Additionally, it must be acknowledged that our research used ChatGPT based on GPT-4, which is not a publicly available model. Consequently, the result may not be directly generalizable to other LLMs, especially open-source models like Llama3 (Meta Platforms, Inc), which might have different underlying architectures and training data sets. Moreover, since our study relied on clinical vignettes that were mock scenarios, the potential for bias based on the cases is significant. The lack of real demographic diversity in these vignettes means that the findings may not accurately reflect social or regional nuances, such as ethnicity, prevalence of disease, or cultural practices, that could influence diagnostic outcomes. This limitation suggests a need for careful consideration when applying these AI tools across different geographic and demographic contexts to ensure the findings are appropriately adapted to local populations. This emphasizes the necessity for AI systems to be evaluated in diverse real-world settings to understand their effectiveness comprehensively and mitigate any bias. This distinction is important to consider when extrapolating our study’s findings to other AI systems. Future studies should not only refine AI’s diagnostic reasoning, but also explore the interpretability of its decision-making process, especially when errors occur. ChatGPT should be considered as a supplementary tool in medical diagnosis, rather than a standalone solution. This reinforces the necessity for combined expertise, where AI supports—but does not replace—human clinical judgment. Further research should expand these findings to a wider range of conditions, especially prevalent diseases with significant public health impacts, to thoroughly assess the practical utility and limitations of AI in medical diagnosis.

### Conclusions

Our study contributes valuable evidence for the ongoing discourse on the role of AI in medical diagnosis. This study provides a foundation for future research to explore the extent to which AI can be trained to recognize increasingly complex and atypical presentations, which is critical for its successful integration into clinical practice.

## Supplementary material

10.2196/58758 Multimedia Appendix 1 Differential medical diagnosis list generated by ChatGPT.

10.2196/58758 Multimedia Appendix 2 Transcript of the conversation with ChatGPT and the answers to all the questions.

## Abbreviations

AI: artificial intelligence
C: category
GAI: generative artificial intelligence
GIM: general internal medicine
GPT: generative pre-trained transformer
LLM: large language model
NLP: natural language processing
USMLE: United States Medical Licensing Examination
