# Supplementary material for: Evaluation of ChatGPT-Generated Differential Diagnosis for Common Diseases With Atypical Presentation: Descriptive Research
Source: JMIR Med Educ. 2024 Jun 21;10:e58758. doi: 10.2196/58758 (PMC11199925; doi:10.2196/58758)
Supplement: Multimedia Appendix 1 [file mededu-v10-e58758-s001.docx]

| Case | Rank 1 | Rank 2 | Rank 3 | Rank 4 | Rank 5 |
| --- | --- | --- | --- | --- | --- |
| 1 | Hyperventilation syndrome | Anxiety related to Obsessive-Compulsive Disorder | Functional gastrointestinal disorder | Electrolyte imbalance or metabolic disorder | Neurological disorder |
| 2 | Asthma | Pulmonary embolism | Acute Respiratory Distress Syndrome | Psychogenic dyspnea | Complications related to hormone therapy |
| 3 | Chronic patulous Eustachian tube | Ménière's disease | Somatic symptoms due to mental stress | Somatic auditory syndrome | Middle ear disease |
| 4 | Irritable Bowel Syndrome | Chronic infection | Drug-induced intestinal disorder | Chronic enteritis | Renal dysfunction |
| 5 | Infectious mononucleosis | Acute hepatitis | Other viral infections | Drug-induced hepatitis | Autoimmune hepatitis |
| 6 | Infectious mononucleosis | Acute pharyngitis | Acute tonsillitis | Viral infections such as rubella or measles | Acute HIV infection |
| 7 | Stroke (especially cerebral infarction or Transient Ischemic Attack) | Alcohol-related neurological disorders | Alcohol withdrawal syndrome | Cerebral hemorrhage | Hypoglycemia |
| 8 | Cervical disc herniation | Cervical spondylosis | Transient Ischemic Attack | Neck muscle pain and nerve compression | Carotid artery dissection |
| 9 | Cervical disc disease | Cervical spondylosis | Radiculopathy | Frozen shoulder (Adhesive capsulitis) | Diabetic neuropathy |
| 10 | Cardiogenic shock | Anemia | Anemia due to chronic disease | Anemia due to drug side effects | Refusal of rectal examination |
| 11 | Infection at the surgical site | Bacterial tenosynovitis | Bacterial arthritis | Cellulitis | NA |
| 12 | Abdominal wall pain syndrome | NA | NA | NA | NA |
| 13 | Polymyositis or Dermatomyositis | Viral infection | Sepsis | Drug side effects | NA |
| 14 | Acute appendicitis | Peptic ulcer | Intestinal obstruction | Gynecological disorders | NA |
| 15 | Menopausal disorders | Cardiac disease | Anxiety disorder | Sleep disorders | NA |
| 16 | Stroke | Transient Ischemic Attack | Brain tumor or metastasis | Encephalitis or meningitis | Drug side effects |
| 17 | Recurrent stroke | Fibromuscular dysplasia | Vertebral artery dissection | Conditions related to weight loss and loss of appetite | NA |
| 18 | Parkinson's disease | Rheumatoid arthritis | Depression | Endocrine disorders | NA |
| 19 | Major Depressive Disorder | Adjustment disorders | Bipolar disorder (Manic depression) | NA | NA |
| 20 | Chronic motion sickness (Mal de debarquement) | Psychogenic vomiting | Dysautonomia | Hyperthyroidism | Gastrointestinal disorders |
| 21 | Gastrointestinal disorders | Myofascial back pain | Lumbar disc disease | Early spinal disease | Secondary back pain due to internal diseases |
| 22 | Iron deficiency anemia | Chronic inflammatory diseases | Pain and instability due to osteoarthritis of the knee | Systemic symptoms due to anemia | NA |
| 23 | Urinary tract infection (especially pyelonephritis) | Enteritis | Complications due to ureteral stones | Colitis or colon problems | NA |
| 24 | Heart failure | Renal dysfunction | Lower extremity venous insufficiency | Side effects due to medications | Malnutrition or protein deficiency |
| 25 | Infective endocarditis | Viral myositis (including epidemic myalgia) | Sepsis | Rheumatic diseases | NA |

NA: not available
