# Supplementary material for: Evaluation of ChatGPT-Generated Differential Diagnosis for Common Diseases With Atypical Presentation: Descriptive Research
Source: JMIR Med Educ. 2024 Jun 21;10:e58758. doi: 10.2196/58758 (PMC11199925; doi:10.2196/58758)
Supplement: Multimedia Appendix 2 [file mededu-v10-e58758-s002.docx]

**Question**:

*“List of differential diagnoses in order of likelihood, based on the provided vignettes.”*

**Answer**:

Case 1:

1. Hyperventilation Syndrome: Patients with a history of hyperventilation syndrome may experience symptoms such as nausea, vomiting, epigastric discomfort, and tingling in the hands and feet caused by hyperventilation. Hyperventilation can lead to a decrease in the blood carbon dioxide concentration, causing hypocapnia. This may result in symptoms such as tingling and numbness in the hands and feet, as well as muscle cramps.
2. Anxiety Associated with Obsessive-Compulsive Disorder (OCD): Paroxetine is a medication used in the treatment of obsessive-compulsive disorder, and the patient's psychological stress may influence somatic symptoms. Anxiety and stress can cause symptoms such as indigestion, nausea, vomiting, and autonomic symptoms (e.g., tingling in the hands and feet).
3. Functional Gastrointestinal Disorders: Nausea, vomiting, and epigastric discomfort are characteristic symptoms of functional gastrointestinal disorders. These include irritable bowel syndrome (IBS) and functional dyspepsia, but tingling in the hands and feet is not typically included in these diagnoses.
4. Electrolyte Imbalance or Metabolic Abnormalities: Tingling and numbness in the hands and feet may also be caused by imbalances in electrolytes such as calcium, magnesium, and potassium. Given the slightly low blood pressure, other metabolic abnormalities such as dehydration or endocrine disorders should also be considered.
5. Neurological Disorders: While the possibility of tingling in the limbs being due to neurological causes cannot be ruled out, the absence of abnormal physical findings makes this possibility less likely. However, neurological symptoms may appear initially if certain conditions or diseases are in their early stages.

Case 2:

1. Exacerbation of Asthma: Given the patient's history of childhood asthma, acute exacerbation of asthma is the most likely cause of respiratory distress. Asthma exacerbations can be triggered by environmental factors, infections, stress, among other factors. While there is documentation of normal lung sounds, wheezing (a high-pitched whistling sound) is commonly heard during asthma attacks. However, in severe cases, lung sounds may become diminished.
2. Pulmonary Embolism (PE): With a SpO2 of 89% and a heart rate of 110 beats per minute, pulmonary embolism is also a possibility. However, the absence of thrombi in the pulmonary veins on contrast-enhanced CT imaging reduces the likelihood of this diagnosis. Pulmonary embolism typically occurs when a clot obstructs the pulmonary artery, causing chest pain, shortness of breath, and sometimes coughing or hemoptysis.
3. Acute Respiratory Distress Syndrome (ARDS): Respiratory distress and low SpO2 are characteristic, but since there are no obvious abnormalities in the lung fields on chest X-ray, this diagnosis relies on other clinical findings. ARDS is a condition characterized by severe inflammation in the lungs, resulting in significant impairment of gas exchange.
4. Psychogenic Respiratory Distress: When no physical cause is found, psychogenic respiratory distress is considered. Psychological stressors such as anxiety or panic disorders can lead to respiratory distress, although a decrease in SpO2 is not typical.
5. Complications Related to Hormonal Therapy: The use of drospirenone and ethinyl estradiol tablets (Yaz) may rarely increase the risk of thrombosis. However, since no thrombus was identified on contrast-enhanced CT, the likelihood of this possibility is low. Nonetheless, continued vigilance regarding the association between hormonal therapy and respiratory distress is warranted.

Case 3:

1. Chronic Eustachian Tube Dysfunction: Experiencing ear fullness and a ringing sensation known as "tinnitus," these symptoms may result from the Eustachian tube failing to close properly. Chronic Eustachian tube dysfunction can cause abnormal sensations of ringing in the ears and hearing disturbances, although audiograms indicate normal hearing.
2. Ménière's Disease: With a history of sudden hearing loss and current complaints of ear fullness and tinnitus, Ménière's disease typically presents with low-frequency hearing loss, tinnitus, vertigo, and ear fullness. However, since audiograms show normal hearing, the likelihood of Ménière's disease in this case may be low.
3. Physical Symptoms Due to Psychological Stress: Given a history of hyperventilation or panic attacks during work and insomnia, symptoms may be related to psychological stress. Stress and anxiety can induce sensations of ear fullness and tinnitus, which may manifest as a form of somatic symptom disorder.
4. Somatosensory Tinnitus Syndrome: Despite no apparent abnormalities in the inner ear or auditory pathways, this condition involves perceiving tinnitus. If tinnitus is associated with psychological stress or a sense of restlessness, somatosensory tinnitus syndrome may also be considered.
5. Potential Middle Ear Disorders: Although no abnormalities are observed in the inner ear and audiograms indicate normal hearing, problems in the middle ear are still plausible. For instance, conditions like otitis media or serous otitis media can cause sensations of ear fullness, although typical signs of otitis media are not reported in this case.

Case 4:

1. Irritable Bowel Syndrome (IBS): Previously diagnosed with "suspected irritable bowel syndrome," the presence of eosinophilic infiltration on biopsy of the ileocecal region supports this diagnosis. IBS can worsen due to nonspecific triggers like stress or dietary changes, but the patient reports no specific stress.
2. Chronic Infection: Although reported as Clostridioides difficile toxin and antigen negative, other infections (e.g., parasitic infections) should be considered as potential causes of chronic diarrhea. The presence of jejunal edema on contrast-enhanced CT suggests infection or inflammatory bowel disease, although stool parasites or steatorrhea are not observed.
3. Drug-induced Enteropathy: The patient has been taking multiple medications for an extended period, which could induce or exacerbate diarrhea. Medications for hypertension or constipation treatment may particularly affect bowel function.
4. Chronic Enteritis: The presence of eosinophilic infiltration suggests the possibility of chronic enteritis (e.g., eosinophilic enteritis), causing chronic inflammation of the intestines and recurrent diarrhea and abdominal pain.
5. Renal Dysfunction: Elevated renal function (BUN 52.8 mg/dL, Cre 2.41 mg/dL) suggests chronic dehydration or direct impact on renal function. Chronic diarrhea can lead to dehydration, potentially causing renal dysfunction.

Case 5:

1. Infectious Mononucleosis: Features include fever, fatigue, scattered erythematous rash on the trunk and limbs, bilateral posterior cervical lymphadenopathy with tenderness, splenomegaly, significant elevation of AST and ALT, and the presence of atypical lymphocytes. These clinical findings strongly suggest infectious mononucleosis, typically caused by acute viral infection, usually by the Epstein-Barr virus (EBV).
2. Acute Hepatitis: Elevated liver enzymes such as AST 530 U/L and ALT 353 U/L suggest acute hepatitis, but with negative HBs antigen and HCV antibodies, the possibility of viral hepatitis is low. Infectious mononucleosis can also cause liver function abnormalities.
3. Other Viral Infections: COVID-19 and influenza tests were negative, but the possibility of other viral infections should be considered. Examples include human immunodeficiency virus (HIV), other respiratory viruses, herpes viruses, although HIV test is negative.
4. Drug-induced Hepatitis: Given the absence of regular medication or supplement use in the current patient, this possibility is low.
5. Autoimmune Hepatitis: Absence of elevation in IgA, IgG, IgM, negative antinuclear antibodies, and no complement deficiency suggests a low likelihood of autoimmune hepatitis.

Case 6:

1. Infectious Mononucleosis: Symptoms such as fever, sore throat, generalized fatigue, loss of appetite, joint pain, night sweats, and swelling of lymph nodes in the neck and under the ears are typical of infectious mononucleosis. Infectious mononucleosis is an acute infection often caused by the Epstein-Barr virus (EBV), particularly common in young adults.
2. Acute Pharyngitis: Fever and sore throat are typical symptoms of acute pharyngitis. However, when accompanied by swollen lymph nodes and systemic symptoms, it suggests a condition beyond simple acute pharyngitis.
3. Acute Tonsillitis: Given the chief complaints of sore throat and fever, the possibility of acute tonsillitis should also be considered. Visual assessment of the tonsils and, if necessary, throat culture can be useful for evaluation.
4. Viral Infections such as Rubella or Measles: Considering the fever, lymph node swelling, and systemic symptoms, the possibility of viral infections like rubella or measles should be considered. While these often present with characteristic rashes, the absence of rash description necessitates diagnosis based on other clinical signs.
5. Initial HIV Infection: Considering travel history, social alcohol consumption, and smoking history, the possibility of initial HIV infection cannot be ruled out. Initial HIV infection can cause nonspecific symptoms such as fever, sore throat, and lymph node swelling.

Case 7:

1. Stroke (particularly ischemic stroke or transient ischemic attack): With a history of atrial fibrillation and despite anticoagulant therapy (warfarin), the risk of stroke increases. Additionally, thrombus formation due to arrhythmia may impede cerebral blood flow. Signs of stroke, such as weakness in the right upper limb (graded 4/5), dysarthria during conversation, and higher cognitive dysfunction (impaired time and place orientation), are evident.
2. Alcohol-related neuropathy: Prolonged excessive alcohol consumption can lead to dysfunction of the nervous system. Symptoms such as altered consciousness, weakness, falls, muscle weakness, and dysarthria may indicate alcohol-related neuropathy.
3. Alcohol withdrawal syndrome: Given the patient's habitual heavy alcohol intake, symptoms such as altered consciousness, tremors, and weakness may be present as withdrawal symptoms. These can occur when alcohol intake is abruptly reduced or stopped.
4. Cerebral hemorrhage: With a history of hypertension and taking warfarin, the risk of cerebral hemorrhage is elevated. However, while altered consciousness and some neurological examination results may suggest cerebral hemorrhage, direct evidence from the examination findings is limited.
5. Hypoglycemia: Irregular eating habits and alcohol intake may lead to hypoglycemia. While hypoglycemia can also cause altered consciousness and weakness, considering other neurological findings, it becomes a lower priority in this case.

Case 8:

1. Cervical disc herniation: Sudden intense pain from the neck to the shoulder and radiating pain to both upper limbs are typical symptoms of cervical disc herniation. In this condition, protrusion of the disc compresses the nerve roots, causing pain and paralysis. The temporary improvement in symptoms suggests that the compression on the nerve root may have been transient.
2. Cervical spondylosis: Degeneration of the cervical spine can lead to compression of nerve roots or the spinal cord, causing neck pain and paralysis in one upper limb. Considering the patient's age of 70 years, cervical spondylosis should be particularly considered.
3. Transient ischemic attack (TIA): Temporary paralysis of the right upper limb suggests the possibility of a TIA. However, neck pain is not a typical symptom of TIA. TIA is a condition where blood flow to the brain temporarily decreases, and symptoms usually resolve within minutes to an hour.
4. Neck muscle pain and nerve compression: Neck muscle pain or tension can compress nerves, causing temporary paralysis. The sudden onset of pain upon awakening suggests that improper posture during sleep may also be a contributing factor.
5. Carotid artery dissection: Severe neck pain and transient paralysis may indicate carotid artery dissection. Carotid artery dissection occurs when the inner lining of the blood vessel tears, allowing blood to enter the vessel wall, reducing blood flow to the brain and potentially causing transient paralysis and other neurological symptoms.

Case 9:

1. Cervical discopathy: The patient experiences numbness and pain from the neck to the upper arm, and mild disc herniation was confirmed on X-ray examination. Allodynia in the C6 to C8 dermatomes indicates that the nerve roots associated with these areas are affected. Despite negative results on cervical nerve root provocation tests, compression or irritation of the nerve roots due to disc herniation may still be the cause.
2. Cervical spondylosis: Being a 70-year-old female, there is a possibility of advanced degeneration of the cervical spine. Cervical spondylosis may involve not only degeneration of the intervertebral discs but also deformity of joints and formation of bone spurs, which can compress nerve roots and cause pain and numbness.
3. Radiculopathy: Pain and numbness from the neck to the arm suggest the possibility of radiculopathy, which occurs when specific nerve roots are compressed or irritated. Given the presence of disc herniation on X-ray examination, radiculopathy is highly likely.
4. Frozen shoulder (adhesive capsulitis): If the pain is localized to the right shoulder, frozen shoulder is a possibility. However, since the distribution of pain matches the C6 to C8 dermatomes in this case, the likelihood of frozen shoulder is low.
5. Diabetic neuropathy: With a history of diabetes, there is a possibility of pain and numbness due to diabetic neuropathy. However, considering that the pain is distributed to specific dermatomes, cervical issues are considered more relevant in this case.

Case 10:

1. Cardiogenic shock: Hypotension (blood pressure 94/40 mmHg), irregular pulse (atrial fibrillation), and pallor of the conjunctiva suggest the possibility of cardiogenic shock. However, the negative troponin I and the absence of typical evidence of acute myocardial infarction indicate otherwise. The presence of atrial fibrillation can lead to mismatches in cardiac blood flow, potentially compromising adequate blood supply to the body.
2. Anemia: A low hemoglobin level of 8.4 g/dL indicates the presence of anemia. Normocytic anemia may result from nutritional deficiencies, chronic illnesses, or bone marrow suppression. Anemia can cause symptoms such as dizziness and pallor.
3. Anemia due to chronic conditions: The presence of chronic conditions such as a history of breast cancer and atrial fibrillation may be the cause of anemia due to chronic illness. These conditions can lead to impaired iron utilization and decreased red blood cell production in the bone marrow.
4. Anemia due to drug side effects: The use of anticoagulants such as rivaroxaban increases the risk of bleeding. Even in the absence of bleeding, detailed examinations are necessary to rule out the possibility of occult bleeding.
5. Refusal of rectal examination: The refusal of a rectal examination means that direct confirmation of gastrointestinal bleeding is not possible. Occult gastrointestinal bleeding could be a cause of anemia.

Case 11:

1. Surgical site infection: Following the incision of the mucous cyst in the left middle finger DIP joint, there is overall swelling extending from the surgical site to the DIP-PIP joint, with a dark red coloration, strongly suggesting bacterial infection at the surgical site. Increased pain, redness, and swelling indicate the progression of the infection.
2. Bacterial tenosynovitis: Bacterial infection of the tendon sheath may occur, especially if accompanied by restricted range of motion at the joint of the finger. This can be a complication particularly after incisional surgery and requires prompt treatment.
3. Bacterial arthritis: Restricted range of motion at the joint suggests the possibility of bacterial arthritis due to infection within the joint. This can occur postoperatively and is caused by bacterial infection within the joint.
4. Cellulitis: Inflammation due to infection of the skin and subcutaneous tissue around the surgical site and its vicinity, characterized by redness, swelling, and warmth. In this scenario, there is a possibility of the infection spreading outward from the surgical site.

Case 12:

1. The diagnosis to consider in this 78-year-old female patient's case is abdominal wall pain syndrome. Point tenderness is observed in the region of the lateral border of the rectus abdominis (Th11), with a positive pinch sign and positive Carnett's sign, strongly suggesting this diagnosis. When Carnett's sign is positive, it indicates that the source of the pain is likely in the abdominal wall rather than in the viscera, suggesting a higher likelihood of issues with the muscles or fascia rather than internal organs.

Case 13:

1. Polymyositis or Dermatomyositis: Generalized muscle pain, fever, and elevated CRP and WBC are observed. These symptoms are consistent with typical features of polymyositis or dermatomyositis. However, since specific skin rashes characteristic of dermatomyositis have not been reported, the likelihood of polymyositis may be higher.
2. Viral infection: Rapid tests for novel coronavirus and influenza viruses were negative, but other viral infections could be the cause of muscle pain and fever. For example, enteroviruses and echoviruses are possible considerations.
3. Sepsis: Elevated CRP levels, fever, and fatigue suggest the possibility of sepsis. However, since typical signs of sepsis (such as conjunctival congestion, jaundice, or a clear source of infection) are not present, this diagnosis remains uncertain.
4. Drug-related side effects: Consideration should also be given to muscle pain and general malaise due to the use of over-the-counter cold remedies. Some cold remedies may contain ingredients that can cause muscle pain and fatigue.

Case 14:

1. Acute Appendicitis: Strong suspicion of acute appendicitis arises from tenderness in the right lower abdomen to the umbilical region. The clinical course, starting with nausea followed by abdominal pain moving from periumbilical to the right lower abdomen, follows a typical pattern. Acute appendicitis is one of the most common acute abdominal conditions in this age group.
2. Peptic Ulcer Disease: Given the epigastric pain and temporary relief with vomiting, peptic ulcer disease is also considered. However, the specific tenderness in the right lower abdomen to the umbilical region may not support this diagnosis.
3. Intestinal Obstruction: Recurrence of ileus is considered due to the history of ileus. Intestinal obstruction can cause abdominal pain, vomiting, and abdominal distension, but there is some doubt about this diagnosis due to the description of a flat and soft abdomen.
4. Gynecological Disorders: Gynecological disorders, especially ovarian torsion or rupture of an ovarian cyst, can also cause abdominal pain. However, with the statement in the referral letter that "there is 100% no possibility of pregnancy," and the localization of tenderness to the right lower abdomen to the umbilical region, the likelihood of acute appendicitis is considered higher in this case.

Case 15:

1. Menopausal Syndrome: Given the report of the last menstruation being six months ago, there's a possibility of symptoms related to menopause. Menopausal syndrome can cause various physical and psychological symptoms due to changes in hormone balance. However, discomfort in the epigastric region and difficulty breathing during sleep are not typical symptoms of menopausal syndrome.
2. Cardiac Conditions: The onset of discomfort in the epigastric region during a walk, followed by improvement after rest, suggests cardiac conditions such as angina. However, since there were no abnormalities found in the electrocardiogram, blood tests, or chest X-ray, the possibility of cardiac conditions may be low. Nevertheless, more detailed cardiac examinations (e.g., exercise stress test, echocardiogram) are necessary to completely rule out cardiac conditions.
3. Anxiety Disorder: Since no abnormalities were found in medical examinations regarding difficulty breathing during sleep and discomfort in the epigastric region, these symptoms could be due to anxiety or stress. In anxiety disorders, physical symptoms may precede psychological ones, especially when health-related anxieties exacerbate these symptoms.
4. Sleep Disorders: Considering the main complaints of difficulty sleeping and breathing during sleep, the possibility of sleep disorders such as sleep apnea syndrome is also considered. While the BMI is within the normal range, sleep disorders are not necessarily directly linked to BMI, so a detailed evaluation of sleep patterns during sleep is necessary.

Case 16:

1. Stroke: The presence of right-sided upper and lower limb paralysis and dysarthria strongly suggests a left hemisphere stroke. Since no brain hemorrhage was observed on the head CT, the likelihood of an ischemic stroke (stroke) is even higher. Additionally, the use of imatinib for GIST treatment may affect blood clotting, which could further increase the risk of stroke.
2. Transient Ischemic Attack (TIA): TIAs cause symptoms similar to stroke but resolve completely within 24 hours. If the patient's symptoms resolve completely within a short period, TIA is a possible diagnosis. However, considering the persistent symptoms at the time of presentation, the likelihood of stroke is higher.
3. Brain Tumor or Metastasis: Given the patient's history of GIST and peritoneal metastasis, the possibility of brain metastasis should be considered. However, since no abnormalities other than bleeding were observed on the initial head CT, the likelihood of brain tumor or metastasis at this point is considered low. Further detailed imaging such as MRI may be necessary.
4. Encephalitis or Meningitis: Infections can cause symptoms such as altered consciousness and unilateral paralysis. However, since there were no specific findings on the head CT, and no mention of other typical symptoms (e.g., high fever, headache, neck stiffness), the likelihood in this case is low.
5. Medication Side Effects: Anticancer drugs including imatinib can rarely cause neurological side effects. However, in this patient's case, symptoms due to stroke or other causes are much more likely than direct neurological impairment from imatinib.

Case 17:

1. Recurrent Stroke: The presence of a history of stroke and vascular abnormalities due to fibromuscular dysplasia (FMD) increases the risk of recurrent stroke. Confirmation of new stroke findings (right ventral pons and left middle cerebellar peduncle) on MRI suggests that this diagnosis is the most likely.
2. Fibromuscular Dysplasia (FMD): Radiographic evidence of fibromuscular dysplasia-like findings on cervical artery angiography suggests that this is the cause of dissection and stenosis of the vertebral artery. FMD is a condition where blood vessels narrow or dilate due to abnormalities in the middle layer, more common in young women but relatively rare in this patient's age group. However, vascular abnormalities due to FMD are a significant cause of stroke.
3. Vertebral Artery Dissection: Dissection of the left vertebral artery was diagnosed three weeks ago, and now stenosis of the right vertebral artery has been observed. Vertebral artery dissection can cause symptoms such as headache, occipital pain, and visual impairment, potentially leading to stroke. The onset of symptoms in this patient is highly associated with secondary stroke due to vertebral artery dissection.
4. Underlying Pathology Related to Weight Loss and Loss of Appetite: These symptoms suggest underlying metabolic abnormalities, systemic inflammatory diseases, or even malignancies. While these symptoms may not directly correlate with stroke, they should be considered in relation to the patient's overall condition.

Case 18:

1. Parkinson's Disease: Symptoms such as fatigue, loss of appetite, mask-like face, restricted tongue protrusion, and speech impairment are typical of Parkinson's disease. It is a neurodegenerative disorder caused by the loss of dopamine-producing neurons, manifesting not only as motor impairments but also non-motor symptoms (fatigue, gastrointestinal symptoms, etc.).
2. Rheumatoid Arthritis: The possibility of rheumatoid arthritis is also considered due to a family history of the disease in the patient's mother and sister, as well as rheumatoid-like deformities in the fingers. Rheumatoid arthritis is an autoimmune disease characterized by joint inflammation, leading to chronic pain and joint deformities.
3. Depression: The patient has been diagnosed with depression at Hospital C's psychiatry department and is undergoing psychiatric treatment. While depression can be accompanied by physical symptoms such as generalized fatigue and loss of appetite, in this patient's case, the prominence of other physical symptoms may indicate an incomplete diagnosis solely as depression.
4. Endocrine Disorders: Symptoms like fatigue and loss of appetite can also be seen in endocrine disorders such as thyroid disease or adrenal insufficiency. While thyroid enlargement is not observed, further hormonal level testing is necessary to rule out these conditions.

Case 19:

1. Major Depressive Disorder (MDD): The patient exhibits multiple symptoms that meet the diagnostic criteria for MDD, including depressed mood, weight loss due to decreased appetite, early morning awakening, and feelings of fatigue. It has been reported that the symptoms worsened following media coverage of celebrity suicides, indicating reactive depression to external events.
2. Adjustment Disorder: The depressive symptoms as an excessive reaction to stressors (in this case, media coverage of celebrity suicides) are consistent with features of adjustment disorder. The symptoms are directly related to the stressor and have appeared relatively quickly (within 1 week).
3. Bipolar Disorder (Bipolar Disorder): The patient has a history of psychiatric consultation, although details are unclear. There is a possibility of past manic episodes, but there are no reports of direct manic episodes based on current symptoms and past history.

Case 20:

1. Chronic motion sickness (Kinetosickness): Motion sickness is a common condition occurring during travel, characterized by nausea and vomiting. This patient has experienced symptoms since starting commuting by bus or train and has a chronic condition that does not respond to standard treatment (dimenhydrinate salicylate or diprophylline tablets).
2. Psychogenic vomiting: When no physical illness is found, stress or anxiety may be related. Psychological stress resulting from changes in a new job or commuting may cause vomiting. Additionally, the anticipation and anxiety of experiencing nausea and vomiting can exacerbate symptoms.
3. Autonomic dysfunction: Given the increased heart rate and finger tremors, there may be an imbalance in the autonomic nervous system. Autonomic dysfunction can be triggered by stress or changes in the environment, leading to various physical symptoms including nausea and vomiting.
4. Hyperthyroidism: The possibility of hyperthyroidism is considered due to the increased heart rate, weight loss, and finger tremors. This condition is characterized by excessive production of thyroid hormones, resulting in symptoms such as increased heart rate, weight loss, and tremors in the hands.
5. Gastrointestinal disorders: Although no significant abnormalities were found on upper gastrointestinal endoscopy despite nausea and vomiting being the main complaints, the possibility of functional gastrointestinal disorders (such as functional dyspepsia) should also be considered.

Case 21:

1. Functional back pain: Back pain that occurs despite the absence of specific structural abnormalities. The lack of abnormalities on lumbar spine X-rays suggests the possibility of functional back pain, particularly attributed to prolonged sitting in office jobs.
2. Myofascial back pain: Back pain resulting from excessive tension or fatigue in the muscles or fascia of the lower back. Persistent pain even at rest and pain severe enough to awaken at night suggest this diagnosis.
3. Lumbar disc degeneration: Condition where pain arises from degeneration of the intervertebral discs in the lumbar spine. Changes may not be visible on X-rays in the early stages, and problems with the discs can be confirmed with more detailed imaging studies like MRI.
4. Early-stage spinal disorders: Back pain can manifest as an early symptom of inflammatory spinal disorders such as ankylosing spondylitis. Characteristics include worsening pain at night or early in the morning, although abnormalities may not be evident on early X-ray findings.
5. Secondary back pain due to internal medical conditions: Possibility of internal medical conditions such as kidney disease, pancreatitis, or abdominal aortic aneurysm causing back pain. The patient is concerned about visceral illnesses, and these conditions are typically evaluated through imaging studies and blood tests.

Case 22:

1. Iron-deficiency anemia: There is a decrease in Hgb to 8.1 mg/dL and microcytic anemia with an MCV of 78.3%. These are typical findings of iron-deficiency anemia, which may also be associated with chronic inflammation. With CRP elevated to 12.1 mg/dL, there is a possibility of some underlying chronic inflammation.
2. Chronic inflammatory conditions: The elevation of CRP suggests chronic inflammation; however, despite ANA being 80-fold (speckled pattern), the absence of specific autoantibodies (ds-DNA antibody, anti-Sm antibody, anti-SS-A/B antibodies) makes the diagnosis of specific systemic autoimmune diseases (e.g., systemic lupus erythematosus, Sjögren's syndrome) difficult. Nevertheless, the positive ANA indicates the potential for some autoimmune processes.
3. Pain and instability associated with osteoarthritis of the knee: The patient complains of pain due to osteoarthritis of the knee, which is considered one of the causes of instability. Knee pain can affect the patient's walking ability, leading to feelings of instability and unsteadiness.
4. Systemic symptoms due to anemia: Anemia can affect various systems in the body, particularly in older adults, causing symptoms such as dizziness, overall fatigue, and shortness of breath.

Case 23:

1. Urinary tract infection (particularly pyelonephritis): Left lower abdominal pain, fever, and elevated white blood cell count on blood tests (WBC 12,170/μL, Neut 82.6%), along with an increased CRP level (11.97 mg/dL), suggest a urinary tract infection. Additionally, the mild dilation of the left ureter and renal pelvis confirmed on abdominal ultrasound and simple CT supports the presence of an ascending urinary tract infection, especially pyelonephritis. While there is a history of ureteral stones, no stones are currently detected.
2. Enteritis: Considering the left lower abdominal pain and fever, gastrointestinal issues should be considered; however, the absence of vomiting or diarrhea lowers the likelihood. Moreover, the lack of signs of peritoneal irritation in physical examination decreases the possibility of acute abdominal pathology originating from the digestive tract.
3. Complications due to ureteral stones: There is a history of ureteral stones, but no stones are found in the current examination. However, if stones were present, they could progress to a urinary tract infection. Stones can obstruct urine flow, leading to infection.
4. Colitis or colonic issues: The left lower abdomen corresponds to the region where the colon is located, so colitis or other colonic problems should be considered. However, without specific symptoms or test results provided, this possibility is not definitive.

Case 24:

1. Heart failure: Pressure-induced edema in both lower legs is a common symptom of heart failure. However, the normal BNP levels and absence of abnormalities on chest X-ray decrease the likelihood of heart failure. While elevated BNP and pulmonary congestion on chest X-ray are typical findings for diagnosing heart failure, the absence of these does not completely rule out heart failure based on clinical symptoms alone.
2. Renal dysfunction: Renal dysfunction is considered as one of the causes of edema in the elderly. Although detailed test results are not provided, evaluating renal function (serum creatinine, blood urea nitrogen, urine tests) is important.
3. Lower limb venous insufficiency: Common in the elderly, this condition occurs due to incompetent valves in the veins, leading to poor blood return to the lower limbs and causing edema. Pressure-induced edema and difficulty with stair climbing suggest this condition.
4. Drug side effects: Particularly, the use of diuretics or drugs that affect renal function can cause edema. Considering the patient's medication list is necessary to assess these possibilities.
5. Malnutrition or protein deficiency: Especially, a decrease in albumin levels can cause edema. In the elderly, inadequate quality or quantity of food intake may contribute to edema.

Case 25:

1. Infective endocarditis: Symptoms such as fever, body aches, and early diastolic heart murmurs suggest the possibility of infective endocarditis. However, typical signs such as Osler's nodes, Janeway lesions, or petechiae on the nails or conjunctiva are not observed. Infective endocarditis is a condition where bacteria or other microorganisms infect the endocardium or heart valves, leading to inflammation, and the diagnosis is confirmed through blood cultures and echocardiography.
2. Viral myositis (including epidemic myalgia): Symptoms such as muscle pain in the limbs, lower back pain, fever, nausea, and vomiting suggest the possibility of viral infection-induced myositis. Epidemic myalgia may be caused by specific viruses, but identifying the causative virus requires detailed viral and blood tests.
3. Sepsis: Considering the presence of fever and systemic pain, sepsis should also be considered. However, apart from a slight increase in CRP, there is no strong evidence directly suggesting sepsis. Sepsis is usually caused by the spread of infection and is diagnosed through blood cultures.
4. Rheumatic diseases: Despite the presence of systemic pain and fever, the low likelihood of rheumatic diseases is indicated by the absence of joint swelling, tenderness, redness, warmth, or limitation of motion. Rheumatic diseases typically present with obvious joint symptoms and specific blood test results (e.g., RF, anti-CCP antibodies).
